# Remodeling of the m^6^A RNA landscape in the conversion of acute lymphoblastic leukemia cells to macrophages

**DOI:** 10.1038/s41375-022-01621-1

**Published:** 2022-06-09

**Authors:** Alberto Bueno-Costa, David Piñeyro, Carlos A. García-Prieto, Vanessa Ortiz-Barahona, Laura Martinez-Verbo, Natalie A. Webster, Byron Andrews, Nitzan Kol, Chen Avrahami, Sharon Moshitch-Moshkovitz, Gideon Rechavi, Manel Esteller

**Affiliations:** 1 Josep Carreras Leukaemia Research Institute (IJC), Badalona, Barcelona, Catalonia Spain; 2 Centro de Investigacion Biomedica en Red Cancer (CIBERONC), 28029 Madrid, Spain; 3 Barcelona Supercomputing Center (BSC), Barcelona, Catalonia Spain; 4 STORM Therapeutics Ltd, Cambridge, UK; 5 Cancer Research Center and Wohl Institute for Translational Medicine, Chaim Sheba Medical Center, Tel-Hashomer, Israel; 6 Department of Human Genetics and Biochemistry, Sackler Faculty of Medicine, Tel Aviv University, Tel Aviv, Israel; 7 Institucio Catalana de Recerca i Estudis Avançats (ICREA), Barcelona, Catalonia Spain; 8 Physiological Sciences Department, School of Medicine and Health Sciences, University of Barcelona (UB), Barcelona, Catalonia Spain

**Keywords:** Cancer genomics, Acute lymphocytic leukaemia

## To the Editor:

Leukemia cells show an altered transcriptome and proteome that can be associated to many genetic and epigenetic defects. Adding complexity to the biology of transformed cells and its RNA and protein landscape, it has recently been shown that cancer cells also exhibit a distorted pattern of chemical modifications of the RNA molecule [1, 2], the so called epitranscriptome. More than 150 differentially modified nucleotides have been reported in various RNA species, affecting transcript structure, stability, splicing, nuclear export, targeting or translational efficiency [1, 2]. The most abundant internal modification of messenger RNA (mRNA) [3] is the methylation of adenosine (A) in the form of m^6^A, affecting numerous features of RNA activity and metabolism [4]. The identification of an m^6^A eraser, FTO [5], represented the first proof of a reversible mRNA modification and has further stimulated epitranscriptome research in cellular differentiation and carcinogenesis. Since then, the molecular pathways of m^6^A have been carefully dissected revealing that the mark is established by an RNA methyltransferase writer complex with a catalytic subunit, METTL3 and several assistant proteins (METTL14, WTAP, RBM15, KIAA1429, and ZC3H13) [4]. In addition to FTO, m^6^A can also be reversed by ALKBH5 [4]. The m^6^A mark is “read” by m^6^A-binding proteins, such as members of the YTH family (YTHDF1-3 and YTHDC1-2), IGF2BP1-3 proteins, and heterogeneous nuclear ribonucleoproteins (hnRNPs) [4].

In hematopoiesis, the generation of all the different types of mature blood cells from hematopoietic stem cells (HSCs) requires a tight control of RNA activity and disrupted patterns of m^6^A RNA modification and alterations in its associated proteins impair physiological hematopoiesis and are also observed in hematological malignancies [6–8]. In this regard, m^6^A marking was shown to be important for the resolution of the naive state of embryonic stem cells to primed cells, the control of cell fate decisions in early hematopoiesis and the maintainance of hematopoietic stem cell identity and symmetric commitment [8, 9]. Less is known about the role of m^6^A RNA decoration in transition of differentiated stages of more mature hematopoietic cells in physiological and pathological microenvironmental conditions. Examples of hematopoietic cell lineage conversion and plasticity include B-cell lymphomas that transdifferentiate to histiocytic/dendritic cell sarcoma and B-cell acute lymphoblastic leukemia (ALL) patients that escape both antibody treatments and chimeric antigen receptor (CAR) T-cell therapy against CD19 by converting to AML. We have recently identified that myeloid-lineage transdifferentiation is associated with a reconfiguration of the DNA methylation landscape [10]. We now investigated whether such transdifferentiation also involves a switch in the m^6^A RNA epitranscriptome.

**Fig. 1:**
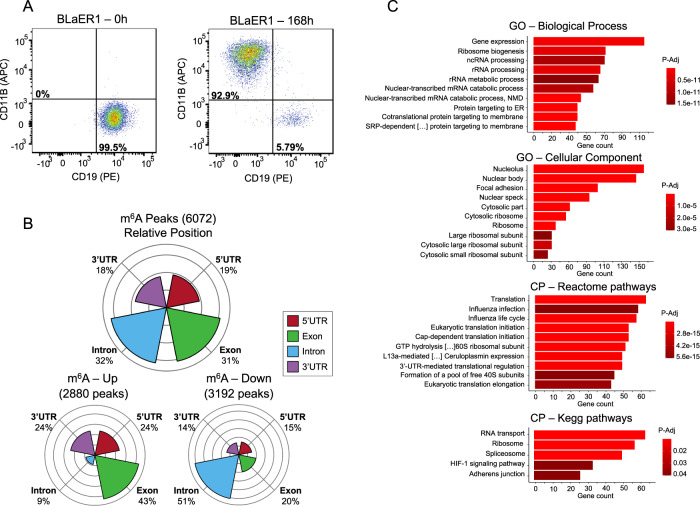
Remodeling of the m^6^A RNA profile in the cellular conversion of pre-B acute lymphoblastic leukemia to macrophages. **A** Flow cytometry plots showing CD11B and CD19 expression with quadrants indicating B cells (CD19+CD11B−) and macrophages (CD19−CD11B+). **B** Distribution of RNA location sites for the m^6^A peaks undergoing changes in the transdifferentiation model. 5′-UTR, 5′-Untranslated Region; 3′-UTR, 3′-Untranslated Region. **C** Gene Ontology (GO) analysis of the genes with distinct m6A content upon pre-B-cell transdifferentiation to macrophage.

Our experimental model involves transdifferentiation of pre-B cells into macrophages. Following early work showing that murine B-cell precursors can be induced by C/EBPα to convert into functional macrophages, we used murine and human cellular models of pre-B cells containing C/EBPα fused with the estrogen receptor hormone-binding domain (C/EBPαER) that transdifferentiate to macrophages upon 17β-estradiol exposure [11, 12]. Primary human BCR-ABL1(+) B-ALL cells can also be reprogrammed into macrophage-like cells by C/EBPα expression [13]. To identify possible remodeling of the m^6^A RNA landscape upon cell conversion, we have herein studied the human precursor B-ALL cell line RCH-ACV transfected with the transgene C/EBPαER, designated below as BLaER1, at the start (0 h) and end (168 h) of transdifferentiation timepoints upon 17β-estradiol exposure, using m^6^A-sequencing (m^6^A-seq) based on immunocapturing and massive parallel sequencing (Supplementary Methods) [14]. The obtained m^6^A-seq raw data have been deposited in the Sequence Read Archive (SRA) BioProject (accession number PRJNA734010).

m^6^A profiling of efficiently transdifferentiated cells, which was validated by the shift in the corresponding CD19 and CD11B markers (Fig. 1A) and additional B-cell and macrophage markers (Fig. S1), revealed that whereas 406 m^6^A peaks (corresponding to 326 transcripts) were stable between the 0 h and 168 h time points, 6072 m^6^A peaks corresponding to 3056 RNA transcripts changed upon cell conversion (Dataset 1). Thus, 94% of the detectable m^6^A peaks changed upon transdifferentiation, indicating that transformation of pre-B ALL cells to macrophages induced profound remodeling of the m^6^A RNA methylome. As to the precise location within the RNA molecule of these m^6^A peaks, 62.5% (3796) were localized in gene-body related sequences, corresponding to 31.6% (1922) and 30.9% (1874) intron and exon RNA sequences, respectively; whereas 19% (1153) were in 5′-untranslated regions (UTRs) and 18.5% (1123) in 3′-UTRs (Fig. 1B). Cell conversion lead to losses in 3192 (52.6%) m^6^A peaks corresponding to 1560 transcripts, and a gain of m^6^A for 2,880 sites (47.4%) corresponding to 1664 transcripts (Fig. 1B). Thus, both m^6^A RNA hypomethylation and hypermethylation events are observed at similar frequencies during transdifferentiation. These results fit with an observed downregulation of both “eraser” and “writer” proteins for the m^6^A mark upon transdifferentiation (Fig. S2).

To further study the identified set of 3056 gene transcripts with significantly distinct m^6^A content upon cell transdifferentiation, we performed functional gene annotation by gene set enrichment analysis (GSEA). We observed an overrepresentation of Gene Ontology (GO) biological processes, molecular function, KEGG pathways and reactome pathways related to RNA ribosome protein translation, such as “ribosome biogenesis”, “ribosomal RNA processing”, “nucleolus”, “translation” and “ribosome”; in addition to RNA regulation (such as “RNA binding” and “RNA transport”) (Fig. 1C) [hypergeometric test with a false discovery rate (FDR) adjusted *p* < 0.05]. Interestingly, when we stratified by up and down m^6^A targets, it was the gain of m^6^A in transdifferention that was associated with GO RNA ribosome protein translation pathways, whereas m^6^A loss was linked to other processes such as transcriptional regulation, splicing and chromatin modifications (Fig. S3). These processes highlight a role for m^6^A as a major contributor to shape the gene expression landscape and, thus, to provide cellular identity.

**Fig. 2:**
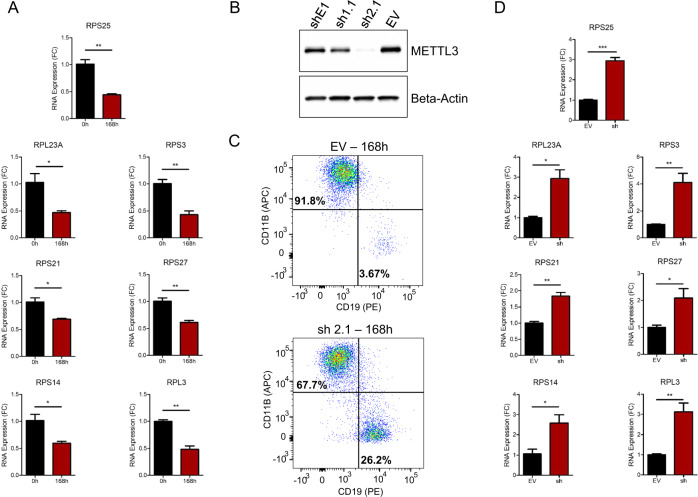
m^6^A mediated downregulation of ribosomal protein genes in transdifferentiation and its restoration upon depletion of the m^6^A RNA methyltransferase MTTL3. **A** RNA expression analyses by qRT-PCR show dowregulation of seven ribosomal protein genes at the end of the cell conversion process (168 h). **B** Western-blot validation of the efficient shRNA-mediated depletion of METTL3 for three clones in BLaER1 cells. EV empty vector. **C** Flow cytometry plots indicate a reduced efficiency of pre-B cell to macrophage transdifferentiation in METTL3 shRNA-depleted cells in comparison to empty vector-transduced cells (no scrambled RNA). **D** qRT-PCR analyses show upregulation of the seven ribosomal protein genes, in comparison to empty vector-transduced cells, at the end of the inefficient cell conversion of METTL3 shRNA-depleted cells.

One key issue related to the impact of m^6^A marks in mRNA function relates to the location of the modification [1, 2]. The first and best-recognized activity of m^6^A is to induce mRNA instability [15], particularly when deposited at 3′-UTRs [1, 2, 4]. To study the effect of identified differential 3′-UTR m^6^A sites on gene expression in our model, we took advantage of the available microarray expression data of the start (0 h) and end (168 h) cell conversion timepoints [12]. We did not observe any overall association between the presence of m^6^A peaks and expression levels (Fisher’s exact test, 2-Tail, *P* value = 0.78), even when stratified for losses and gains of m^6^A vs upregulation or downregulation of the corresponding transcripts (Fisher’s exact test, 2-Tail, *P* value = 0.29) (Fig. S4). We observed, in agreement with the previously published literature [1, 2, 4], that an increase in 3′-UTR m^6^A sites was associated with transcript downregulation, taking into consideration the 742 m^6^A peaks that exhibited a corresponding transcript in the expression microarray (Fisher’s exact test, 2-Tail, *P* value = 0.017). Most importantly, and in agreement with the GO results (Fig. 1C and Fig. S3), we found an enrichment in genes related to protein ribosome translation for these transcripts enriched by m^6^A at their 3′-UTR (Fisher’s *P* value = 1.2 × 10e−13). Among these genes, many ribosomal proteins are some of the most significantly downregulated upon m^6^A 3-UTR increased deposition accompanying the transdifferentiation process (Dataset 2). We further validated by Quantitative Reverse Transcription PCR (qRT-PCR) the seven top genes in this category (RPS25, RPL23A, RPS3, RPS21, RPS27, RPS14, and RPL3) and we confirmed their significant downregulation at the end of the cell conversion (Fig. 2A). Importantly, using the Actinomycin D assay to determine the mRNA stability of our top candidate RSP25, which is downregulated at the RNA (Fig. 2A) and protein (Fig. S5) levels in our model, we observed that transdifferentiation induced a reduction of transcript stability (Fig. S5). This results is in agreement with the proposed role of a gain of m^6^A mark in the 3′-UTR [1, 2, 4]. Interestingly, cells in a middle time point of the conversion process (72 h) show intermediate values for all the above-described parameters (B-cell vs macrophage markers, m^6^A-seq, and ribosomal protein expression patterns) (Fig. S6), supporting that the described transdifferentiation model reflects a gradual change from one cell type to the other.

Having shown the above associations that support a functional role for m^6^A in pre-B cell to macrophage transdifferentiation, particularly targeting 3′-UTRs of ribosome-associated transcripts, we experimentaly validated this model by knocking down the main m^6^A RNA methyltransferase, METTL3 [4]. Efficient shRNA-mediated downregulation of METTL3 in the BLaER1 pre-B cells using three different target sequences (Fig. 2B and Fig. S7) lead to a significant decrease in transdifferentiation rate as measured by B-cell and macrophage markers (Fig. 2C and Fig. S7). The impairment of transdifferentiation was maintained even at 240 h (Fig. S8). Remarkably, the inefficient induction of cell conversion in the METTL3 knockdown model leads to an increase in cell growth and reduced apoptosis that is associated with the predominance of the proliferating B-ALL cells that cannot commit to macrophage transdifferentiation (Fig. S9). Interestingly, the use of STM2457, a METTL3 drug inhibitor [16], mimicked the METTL3 shRNA results by also reducing m^6^A content and impairing transdifferentiation (Fig. S10). Most importantly, METTL3 knockdown cells did not exhibit m^6^A-mediated downregulation of the seven ribosomal proteins observed in our B-cell to macrophage conversion model (Fig. 2A), that instead were upregulated in comparison to the empty-vector-transduced cells (Fig. 2D and Fig. S11). Interestingly, the sorted out population of successfully transdifferentiated CD19-/CD11B+ cells upon METTL3 knockdown showed partial upregulation of ribosomal proteins, 4 of 7 (57%) (Fig. S12). Finally, METTL3 knockdown cells, showing reduction of the overall m^6^A mark, exhibited an increased stability of the RPS25 mRNA transcript according to the Actinomycin D assay (Fig. S13).

Overall, these results indicate a relevant activity of m^6^A RNA marks in the succesfull generation of a macrophage from pre-B cells, particularly by decorating the 3′-UTRs of genes related to the ribosome translational machinery. These findings highlight the role of this epitranscriptomic signal, and the proteins controling and mediating its deposition and downstream effects, in hematological transdifferentiation pathways.

## Supplementary information

Supplementary Figure S1

Supplementary Figure S2

Supplementary Figure S3

Supplementary Figure S4

Supplementary Figure S5

Supplementary Figure S6

Supplementary Figure S7

Supplementary Figure S8

Supplementary Figure S9

Supplementary Figure S10

Supplementary Figure S11

Supplementary Figure S12

Supplementary Figure S13

Dataset S1

Dataset S2
