# Supplementary material for: Remodeling of the m6A RNA landscape in the conversion of acute lymphoblastic leukemia cells to macrophages
Source: Leukemia. 2022 Jun 9;36(8):2121–4. doi: 10.1038/s41375-022-01621-1 (PMC9343246; doi:10.1038/s41375-022-01621-1)
Supplement: Supplementary file 1 — Supplementary Figure S1 [file 41375_2022_1621_MOESM1_ESM.pptx]

## Slide 1
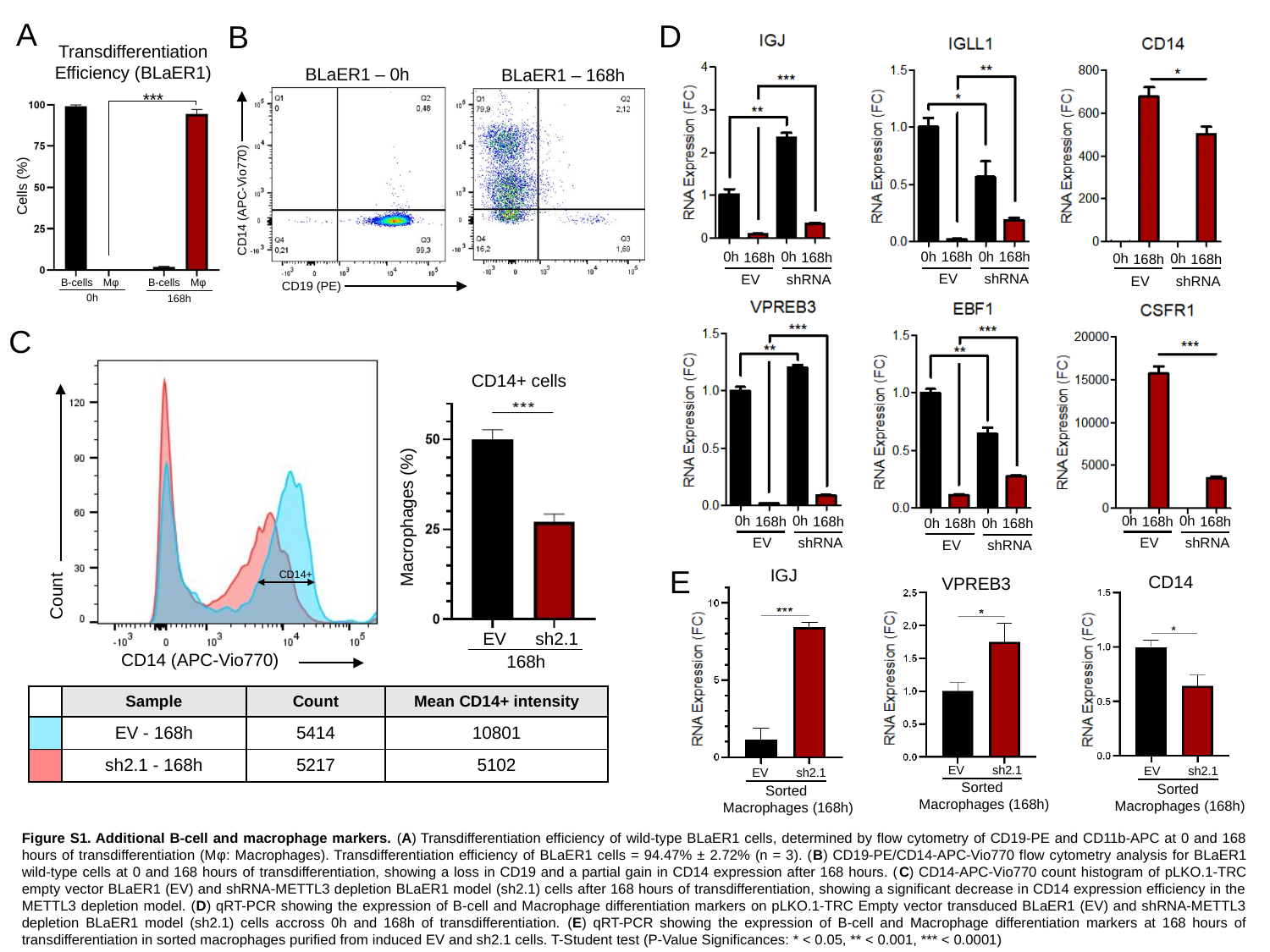

A
D
B
0h
0h
168h
168h
shRNA
EV
0h
0h
168h
168h
shRNA
EV
0h
0h
168h
168h
shRNA
EV
Transdifferentiation
Efficiency (BLaER1)
BLaER1 – 0h
BLaER1 – 168h
CD14 (APC-Vio770)
CD19 (PE)
***
Cells (%)
B-cells
Mφ
B-cells
Mφ
0h
0h
0h
168h
168h
shRNA
EV
168h
0h
0h
168h
168h
shRNA
EV
0h
0h
168h
168h
shRNA
EV
C
CD14+
Count
CD14 (APC-Vio770)
CD14+ cells
Macrophages (%)
VPREB3
EV
sh2.1
Sorted
Macrophages (168h)
IGJ
EV
sh2.1
Sorted
Macrophages (168h)
CD14
EV
sh2.1
Sorted
Macrophages (168h)
E
EV sh2.1
168h
| | Sample | Count | Mean CD14+ intensity |
| --- | --- | --- | --- |
| | EV - 168h | 5414 | 10801 |
| | sh2.1 - 168h | 5217 | 5102 |
Figure S1. Additional B-cell and macrophage markers. (A) Transdifferentiation efficiency of wild-type BLaER1 cells, determined by flow cytometry of CD19-PE and CD11b-APC at 0 and 168 hours of transdifferentiation (Mφ: Macrophages). Transdifferentiation efficiency of BLaER1 cells = 94.47% ± 2.72% (n = 3). (B) CD19-PE/CD14-APC-Vio770 flow cytometry analysis for BLaER1 wild-type cells at 0 and 168 hours of transdifferentiation, showing a loss in CD19 and a partial gain in CD14 expression after 168 hours. (C) CD14-APC-Vio770 count histogram of pLKO.1-TRC empty vector BLaER1 (EV) and shRNA-METTL3 depletion BLaER1 model (sh2.1) cells after 168 hours of transdifferentiation, showing a significant decrease in CD14 expression efficiency in the METTL3 depletion model. (D) qRT-PCR showing the expression of B-cell and Macrophage differentiation markers on pLKO.1-TRC Empty vector transduced BLaER1 (EV) and shRNA-METTL3 depletion BLaER1 model (sh2.1) cells accross 0h and 168h of transdifferentiation. (E) qRT-PCR showing the expression of B-cell and Macrophage differentiation markers at 168 hours of transdifferentiation in sorted macrophages purified from induced EV and sh2.1 cells. T-Student test (P-Value Significances: * < 0.05, ** < 0.001, *** < 0.0001)
