# Supplementary material for: Remodeling of the m6A RNA landscape in the conversion of acute lymphoblastic leukemia cells to macrophages
Source: Leukemia. 2022 Jun 9;36(8):2121–4. doi: 10.1038/s41375-022-01621-1 (PMC9343246; doi:10.1038/s41375-022-01621-1)
Supplement: Supplementary file 2 — Supplementary Figure S2 [file 41375_2022_1621_MOESM2_ESM.pptx]

## Slide 1
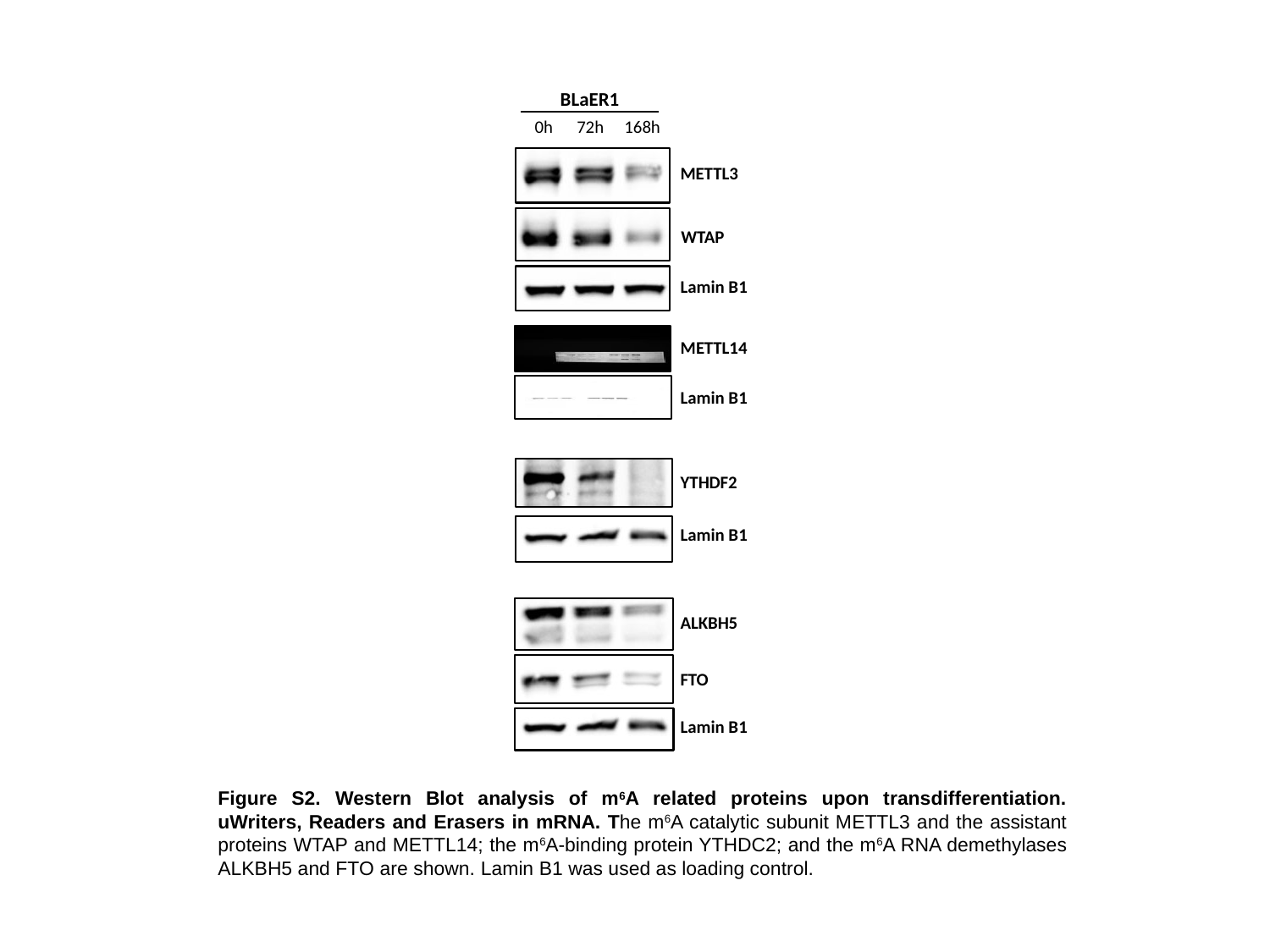

BLaER1
0h
72h
168h
METTL3
WTAP
Lamin B1
METTL14
Lamin B1
YTHDF2
Lamin B1
ALKBH5
FTO
Lamin B1
Figure S2. Western Blot analysis of m6A related proteins upon transdifferentiation. uWriters, Readers and Erasers in mRNA. The m6A catalytic subunit METTL3 and the assistant proteins WTAP and METTL14; the m6A-binding protein YTHDC2; and the m6A RNA demethylases ALKBH5 and FTO are shown. Lamin B1 was used as loading control.
