# Supplementary material for: Remodeling of the m6A RNA landscape in the conversion of acute lymphoblastic leukemia cells to macrophages
Source: Leukemia. 2022 Jun 9;36(8):2121–4. doi: 10.1038/s41375-022-01621-1 (PMC9343246; doi:10.1038/s41375-022-01621-1)
Supplement: Supplementary file 3 — Supplementary Figure S3 [file 41375_2022_1621_MOESM3_ESM.pptx]

## Slide 1
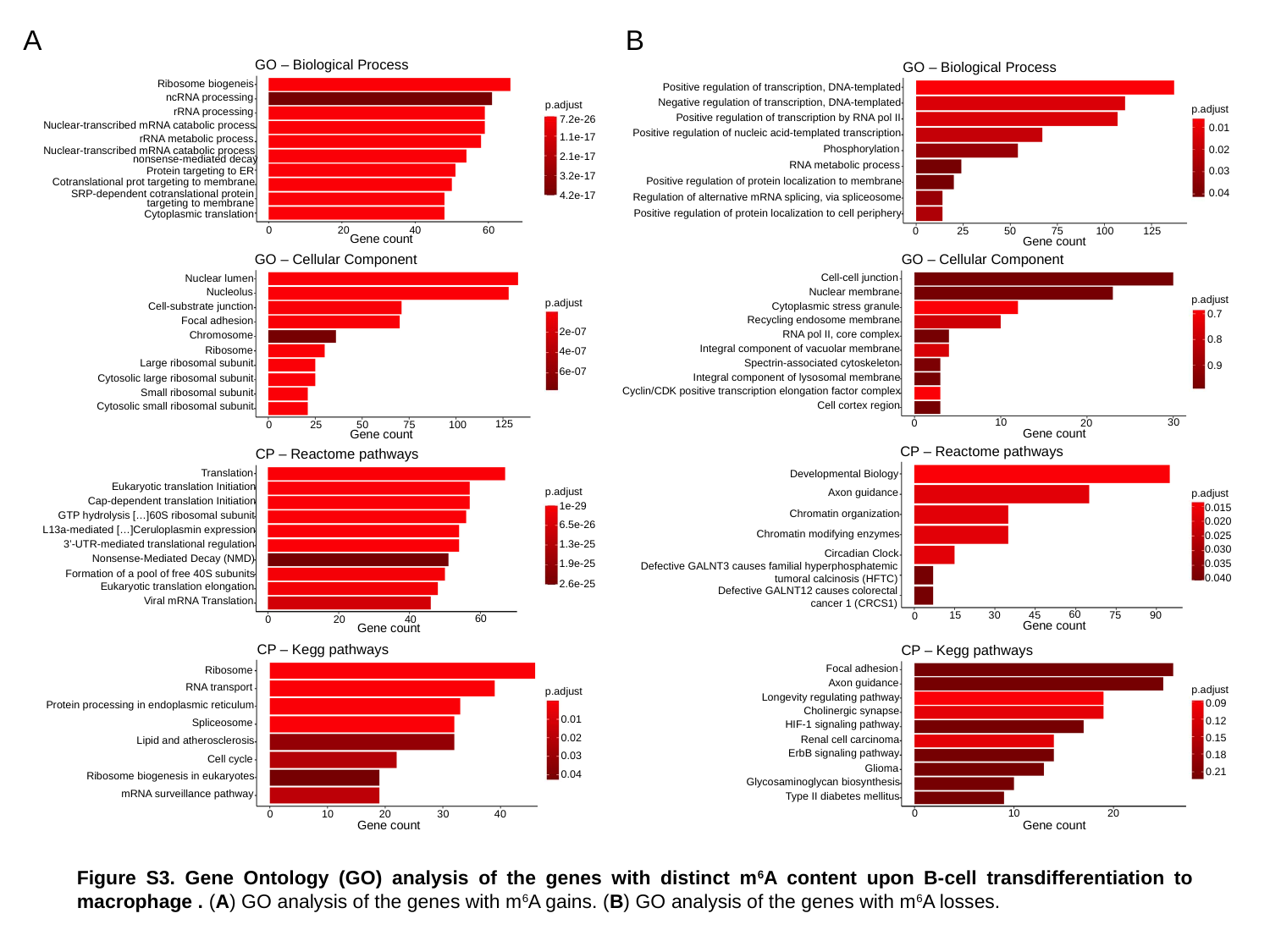

A
B
GO – Biological Process
Ribosome biogeneis
ncRNA processing
p.adjust
7.2e-26
1.1e-17
2.1e-17
3.2e-17
4.2e-17
rRNA processing
Nuclear-transcribed mRNA catabolic process
rRNA metabolic process
Nuclear-transcribed mRNA catabolic process
nonsense-mediated decay
Protein targeting to ER
Cotranslational prot targeting to membrane
SRP-dependent cotranslational protein
targeting to membrane
Cytoplasmic translation
20
40
60
0
Gene count
GO – Cellular Component
Nuclear lumen
Nucleolus
p.adjust
2e-07
4e-07
6e-07
Cell-substrate junction
Focal adhesion
Chromosome
Ribosome
Large ribosomal subunit
Cytosolic large ribosomal subunit
Small ribosomal subunit
Cytosolic small ribosomal subunit
125
0
25
50
75
100
Gene count
CP – Reactome pathways
Translation
Eukaryotic translation Initiation
p.adjust
1e-29
6.5e-26
1.3e-25
1.9e-25
2.6e-25
Cap-dependent translation Initiation
GTP hydrolysis […]60S ribosomal subunit
L13a-mediated […]Ceruloplasmin expression
3’-UTR-mediated translational regulation
Nonsense-Mediated Decay (NMD)
Formation of a pool of free 40S subunits
Eukaryotic translation elongation
Viral mRNA Translation
60
0
20
40
Gene count
CP – Kegg pathways
Ribosome
RNA transport
p.adjust
0.01
0.02
0.03
0.04
Protein processing in endoplasmic reticulum
Spliceosome
Lipid and atherosclerosis
Cell cycle
Ribosome biogenesis in eukaryotes
mRNA surveillance pathway
0
10
20
30
40
Gene count
GO – Biological Process
Positive regulation of transcription, DNA-templated
Negative regulation of transcription, DNA-templated
p.adjust
0.01
0.02
0.03
0.04
Positive regulation of transcription by RNA pol II
Positive regulation of nucleic acid-templated transcription
Phosphorylation
RNA metabolic process
Positive regulation of protein localization to membrane
Regulation of alternative mRNA splicing, via spliceosome
Positive regulation of protein localization to cell periphery
0
25
50
75
100
125
Gene count
GO – Cellular Component
Cell-cell junction
Nuclear membrane
p.adjust
0.7
0.8
0.9
Cytoplasmic stress granule
Recycling endosome membrane
RNA pol II, core complex
Integral component of vacuolar membrane
Spectrin-associated cytoskeleton
Integral component of lysosomal membrane
Cyclin/CDK positive transcription elongation factor complex
Cell cortex region
10
30
20
0
Gene count
CP – Reactome pathways
Developmental Biology
Axon guidance
p.adjust
0.015
0.020
0.025
0.030
0.035
0.040
Chromatin organization
Chromatin modifying enzymes
Circadian Clock
Defective GALNT3 causes familial hyperphosphatemic
tumoral calcinosis (HFTC)
Defective GALNT12 causes colorectal
cancer 1 (CRCS1)
60
90
15
30
45
75
0
Gene count
CP – Kegg pathways
Focal adhesion
Axon guidance
p.adjust
0.09
0.12
0.15
0.18
0.21
Longevity regulating pathway
Cholinergic synapse
HIF-1 signaling pathway
Renal cell carcinoma
ErbB signaling pathway
Glioma
Glycosaminoglycan biosynthesis
Type II diabetes mellitus
10
20
0
Gene count
Figure S3. Gene Ontology (GO) analysis of the genes with distinct m6A content upon B-cell transdifferentiation to macrophage . (A) GO analysis of the genes with m6A gains. (B) GO analysis of the genes with m6A losses.
