# Supplementary material for: Remodeling of the m6A RNA landscape in the conversion of acute lymphoblastic leukemia cells to macrophages
Source: Leukemia. 2022 Jun 9;36(8):2121–4. doi: 10.1038/s41375-022-01621-1 (PMC9343246; doi:10.1038/s41375-022-01621-1)
Supplement: Supplementary file 4 — Supplementary Figure S4 [file 41375_2022_1621_MOESM4_ESM.pptx]

## Slide 1
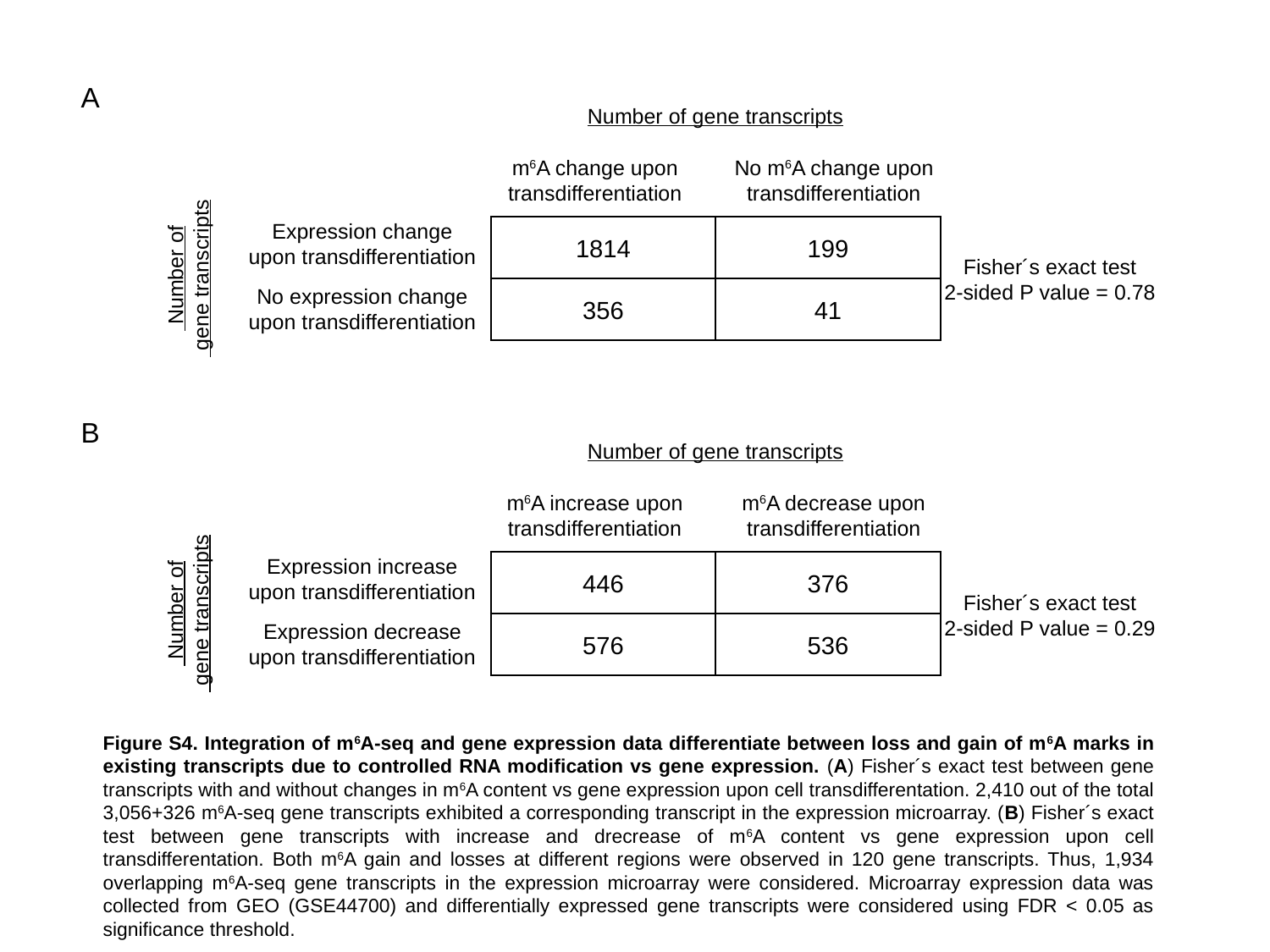

A
Number of gene transcripts
m6A change upon
transdifferentiation
No m6A change upon
transdifferentiation
Expression change
upon transdifferentiation
| 1814 | 199 |
| --- | --- |
| 356 | 41 |
 Number of
 gene transcripts
Fisher´s exact test
2-sided P value = 0.78
No expression change
upon transdifferentiation
B
Number of gene transcripts
m6A increase upon
transdifferentiation
m6A decrease upon
transdifferentiation
Expression increase
upon transdifferentiation
| 446 | 376 |
| --- | --- |
| 576 | 536 |
 Number of
 gene transcripts
Fisher´s exact test
2-sided P value = 0.29
Expression decrease
upon transdifferentiation
Figure S4. Integration of m6A-seq and gene expression data differentiate between loss and gain of m6A marks in existing transcripts due to controlled RNA modification vs gene expression. (A) Fisher´s exact test between gene transcripts with and without changes in m6A content vs gene expression upon cell transdifferentation. 2,410 out of the total 3,056+326 m6A-seq gene transcripts exhibited a corresponding transcript in the expression microarray. (B) Fisher´s exact test between gene transcripts with increase and drecrease of m6A content vs gene expression upon cell transdifferentation. Both m6A gain and losses at different regions were observed in 120 gene transcripts. Thus, 1,934 overlapping m6A-seq gene transcripts in the expression microarray were considered. Microarray expression data was collected from GEO (GSE44700) and differentially expressed gene transcripts were considered using FDR < 0.05 as significance threshold.
