# Supplementary material for: Remodeling of the m6A RNA landscape in the conversion of acute lymphoblastic leukemia cells to macrophages
Source: Leukemia. 2022 Jun 9;36(8):2121–4. doi: 10.1038/s41375-022-01621-1 (PMC9343246; doi:10.1038/s41375-022-01621-1)
Supplement: Supplementary file 5 — Supplementary Figure S5 [file 41375_2022_1621_MOESM5_ESM.pptx]

## Slide 1
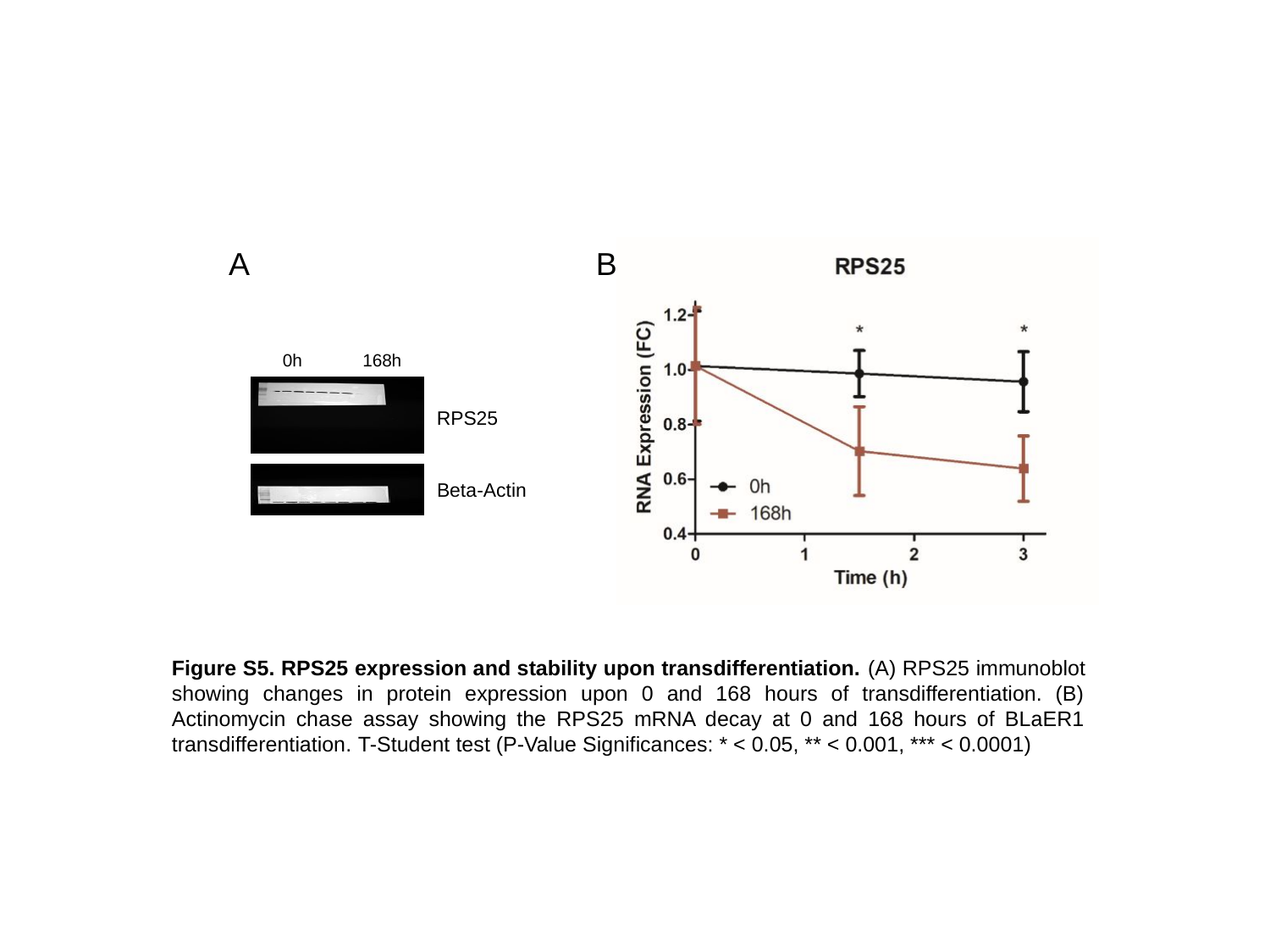

A
B
0h
168h
RPS25
Beta-Actin
Figure S5. RPS25 expression and stability upon transdifferentiation. (A) RPS25 immunoblot showing changes in protein expression upon 0 and 168 hours of transdifferentiation. (B) Actinomycin chase assay showing the RPS25 mRNA decay at 0 and 168 hours of BLaER1 transdifferentiation. T-Student test (P-Value Significances: * < 0.05, ** < 0.001, *** < 0.0001)
