# Supplementary material for: Remodeling of the m6A RNA landscape in the conversion of acute lymphoblastic leukemia cells to macrophages
Source: Leukemia. 2022 Jun 9;36(8):2121–4. doi: 10.1038/s41375-022-01621-1 (PMC9343246; doi:10.1038/s41375-022-01621-1)
Supplement: Supplementary file 6 — Supplementary Figure S6 [file 41375_2022_1621_MOESM6_ESM.pptx]

## Slide 1
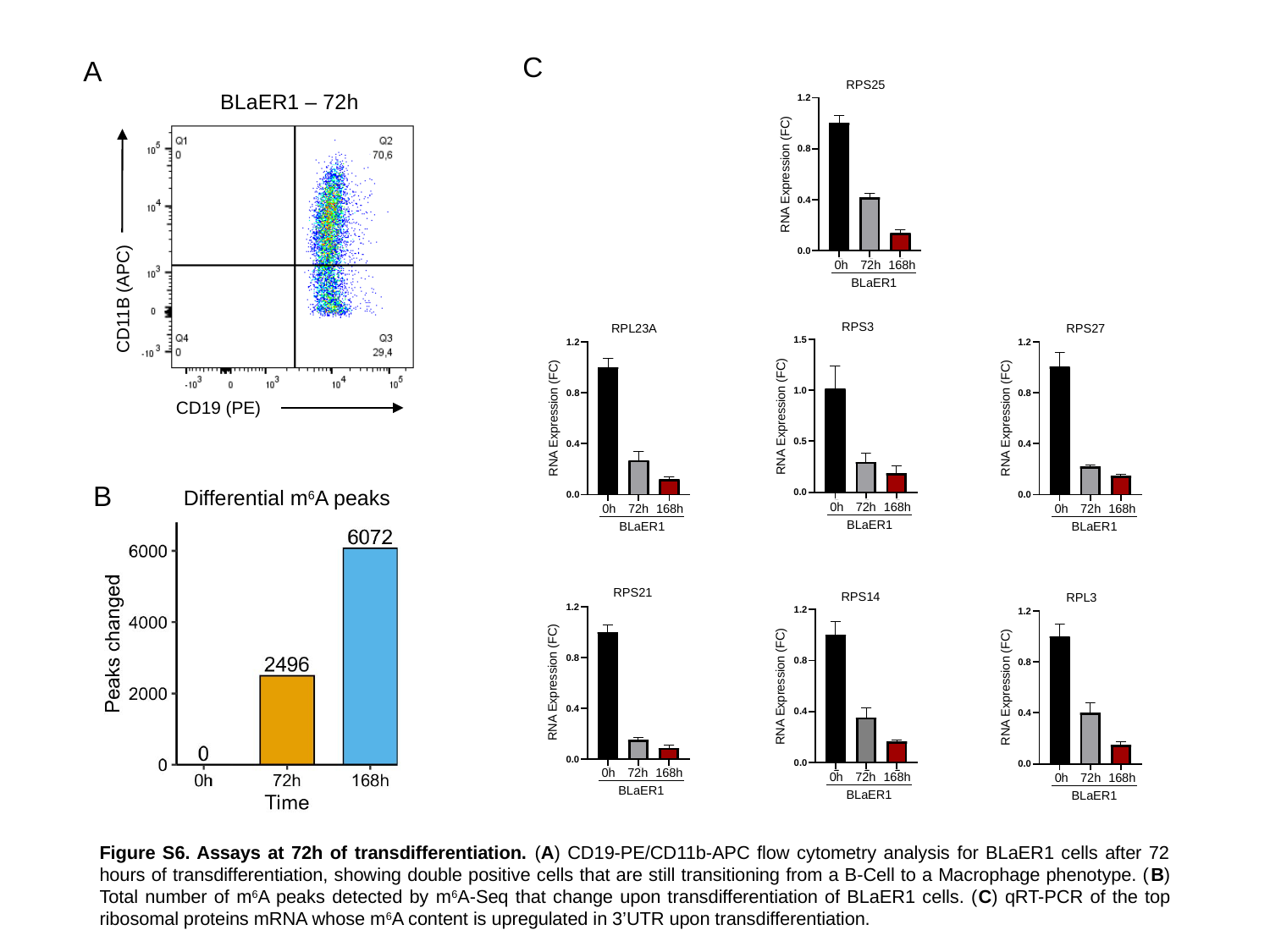

C
A
RPS25
BLaER1 – 72h
RNA Expression (FC)
0h
72h
168h
BLaER1
CD11B (APC)
RPS3
RPL23A
RPS27
CD19 (PE)
RNA Expression (FC)
RNA Expression (FC)
RNA Expression (FC)
B
Differential m6A peaks
0h
72h
168h
0h
72h
168h
0h
72h
168h
BLaER1
BLaER1
BLaER1
RPS21
RPS14
RPL3
RNA Expression (FC)
RNA Expression (FC)
RNA Expression (FC)
0h
72h
168h
0h
72h
168h
0h
72h
168h
BLaER1
BLaER1
BLaER1
Figure S6. Assays at 72h of transdifferentiation. (A) CD19-PE/CD11b-APC flow cytometry analysis for BLaER1 cells after 72 hours of transdifferentiation, showing double positive cells that are still transitioning from a B-Cell to a Macrophage phenotype. (B) Total number of m6A peaks detected by m6A-Seq that change upon transdifferentiation of BLaER1 cells. (C) qRT-PCR of the top ribosomal proteins mRNA whose m6A content is upregulated in 3’UTR upon transdifferentiation.
