# Supplementary material for: Remodeling of the m6A RNA landscape in the conversion of acute lymphoblastic leukemia cells to macrophages
Source: Leukemia. 2022 Jun 9;36(8):2121–4. doi: 10.1038/s41375-022-01621-1 (PMC9343246; doi:10.1038/s41375-022-01621-1)
Supplement: Supplementary file 7 — Supplementary Figure S7 [file 41375_2022_1621_MOESM7_ESM.pptx]

## Slide 1
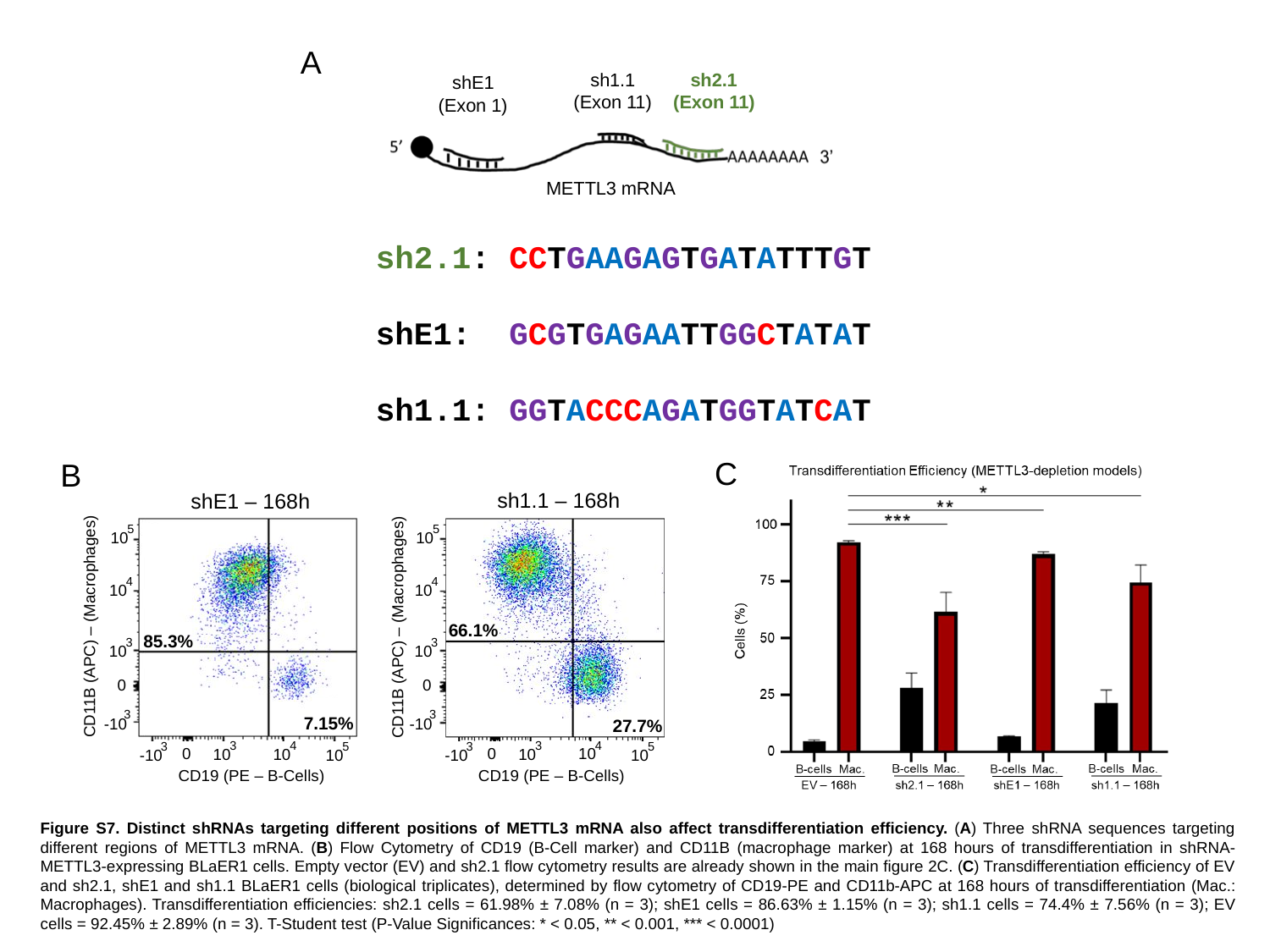

A
sh1.1
(Exon 11)
sh2.1
(Exon 11)
shE1
(Exon 1)
METTL3 mRNA
sh2.1: CCTGAAGAGTGATATTTGT
shE1: GCGTGAGAATTGGCTATAT
sh1.1: GGTACCCAGATGGTATCAT
C
B
sh1.1 – 168h
shE1 – 168h
CD11B (APC) – (Macrophages)
CD19 (PE – B-Cells)
5
10
5
10
4
10
4
10
CD11B (APC) – (Macrophages)
66.1%
85.3%
3
10
3
10
0
0
3
-10
3
-10
7.15%
27.7%
0
0
4
10
4
10
3
10
3
10
3
-10
5
10
3
-10
5
10
CD19 (PE – B-Cells)
Figure S7. Distinct shRNAs targeting different positions of METTL3 mRNA also affect transdifferentiation efficiency. (A) Three shRNA sequences targeting different regions of METTL3 mRNA. (B) Flow Cytometry of CD19 (B-Cell marker) and CD11B (macrophage marker) at 168 hours of transdifferentiation in shRNA-METTL3-expressing BLaER1 cells. Empty vector (EV) and sh2.1 flow cytometry results are already shown in the main figure 2C. (C) Transdifferentiation efficiency of EV and sh2.1, shE1 and sh1.1 BLaER1 cells (biological triplicates), determined by flow cytometry of CD19-PE and CD11b-APC at 168 hours of transdifferentiation (Mac.: Macrophages). Transdifferentiation efficiencies: sh2.1 cells = 61.98% ± 7.08% (n = 3); shE1 cells = 86.63% ± 1.15% (n = 3); sh1.1 cells = 74.4% ± 7.56% (n = 3); EV cells = 92.45% ± 2.89% (n = 3). T-Student test (P-Value Significances: * < 0.05, ** < 0.001, *** < 0.0001)
