# Supplementary material for: Remodeling of the m6A RNA landscape in the conversion of acute lymphoblastic leukemia cells to macrophages
Source: Leukemia. 2022 Jun 9;36(8):2121–4. doi: 10.1038/s41375-022-01621-1 (PMC9343246; doi:10.1038/s41375-022-01621-1)
Supplement: Supplementary file 8 — Supplementary Figure S8 [file 41375_2022_1621_MOESM8_ESM.pptx]

## Slide 1
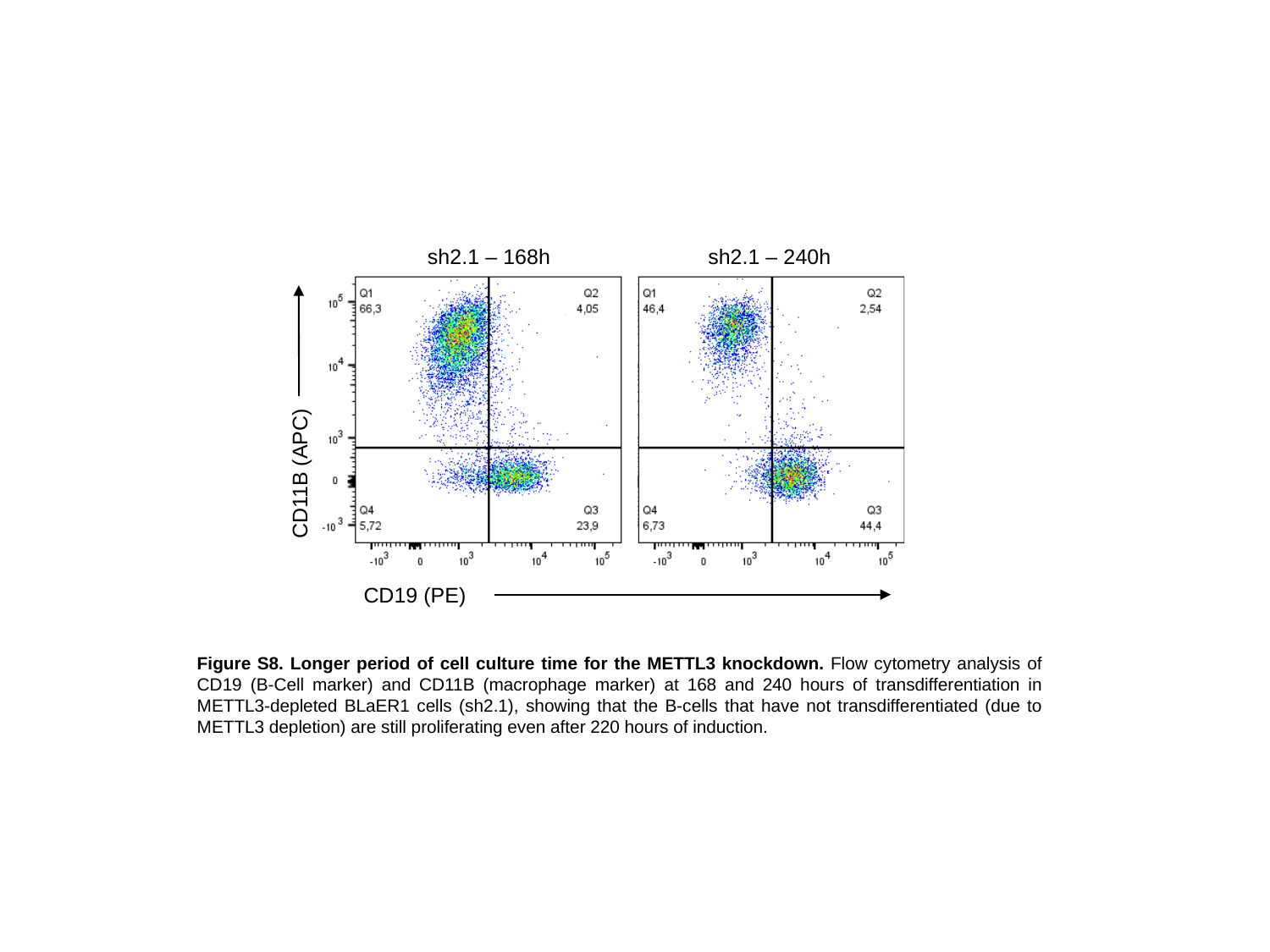

sh2.1 – 240h
sh2.1 – 168h
CD11B (APC)
CD19 (PE)
Figure S8. Longer period of cell culture time for the METTL3 knockdown. Flow cytometry analysis of CD19 (B-Cell marker) and CD11B (macrophage marker) at 168 and 240 hours of transdifferentiation in METTL3-depleted BLaER1 cells (sh2.1), showing that the B-cells that have not transdifferentiated (due to METTL3 depletion) are still proliferating even after 220 hours of induction.
