# Supplementary material for: Remodeling of the m6A RNA landscape in the conversion of acute lymphoblastic leukemia cells to macrophages
Source: Leukemia. 2022 Jun 9;36(8):2121–4. doi: 10.1038/s41375-022-01621-1 (PMC9343246; doi:10.1038/s41375-022-01621-1)
Supplement: Supplementary file 9 — Supplementary Figure S9 [file 41375_2022_1621_MOESM9_ESM.pptx]

## Slide 1
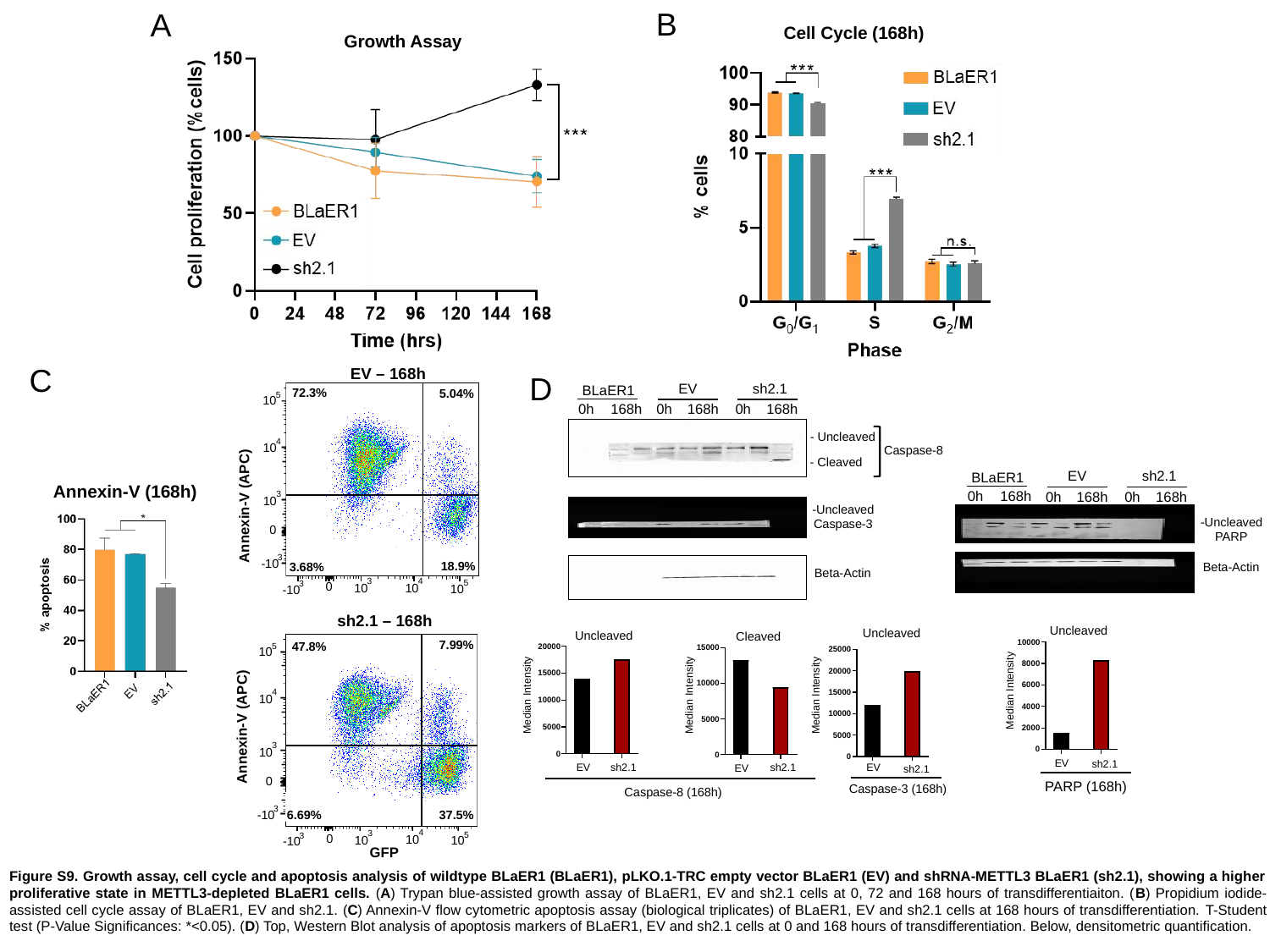

B
A
Cell Cycle (168h)
Growth Assay
C
EV – 168h
D
EV
sh2.1
BLaER1
72.3%
5.04%
5
10
0h
168h
0h
168h
0h
168h
- Uncleaved
4
10
Caspase-8
- Cleaved
EV
sh2.1
BLaER1
0h
168h
0h
168h
0h
168h
-Uncleaved
PARP
Beta-Actin
Annexin-V (168h)
3
10
Annexin-V (APC)
-Uncleaved
Caspase-3
0
3
-10
18.9%
3.68%
Beta-Actin
0
4
10
3
10
5
10
3
-10
sh2.1 – 168h
Uncleaved
Median Intensity
EV
sh2.1
PARP (168h)
Uncleaved
Median Intensity
EV
sh2.1
Caspase-3 (168h)
Uncleaved
Cleaved
Median Intensity
Median Intensity
sh2.1
sh2.1
EV
EV
Caspase-8 (168h)
7.99%
47.8%
5
10
4
10
Annexin-V (APC)
3
10
0
3
-10
6.69%
37.5%
0
4
10
3
10
5
10
3
-10
GFP
Figure S9. Growth assay, cell cycle and apoptosis analysis of wildtype BLaER1 (BLaER1), pLKO.1-TRC empty vector BLaER1 (EV) and shRNA-METTL3 BLaER1 (sh2.1), showing a higher proliferative state in METTL3-depleted BLaER1 cells. (A) Trypan blue-assisted growth assay of BLaER1, EV and sh2.1 cells at 0, 72 and 168 hours of transdifferentiaiton. (B) Propidium iodide-assisted cell cycle assay of BLaER1, EV and sh2.1. (C) Annexin-V flow cytometric apoptosis assay (biological triplicates) of BLaER1, EV and sh2.1 cells at 168 hours of transdifferentiation. T-Student test (P-Value Significances: *<0.05). (D) Top, Western Blot analysis of apoptosis markers of BLaER1, EV and sh2.1 cells at 0 and 168 hours of transdifferentiation. Below, densitometric quantification.
