# Supplementary material for: Remodeling of the m6A RNA landscape in the conversion of acute lymphoblastic leukemia cells to macrophages
Source: Leukemia. 2022 Jun 9;36(8):2121–4. doi: 10.1038/s41375-022-01621-1 (PMC9343246; doi:10.1038/s41375-022-01621-1)
Supplement: Supplementary file 10 — Supplementary Figure S10 [file 41375_2022_1621_MOESM10_ESM.pptx]

## Slide 1
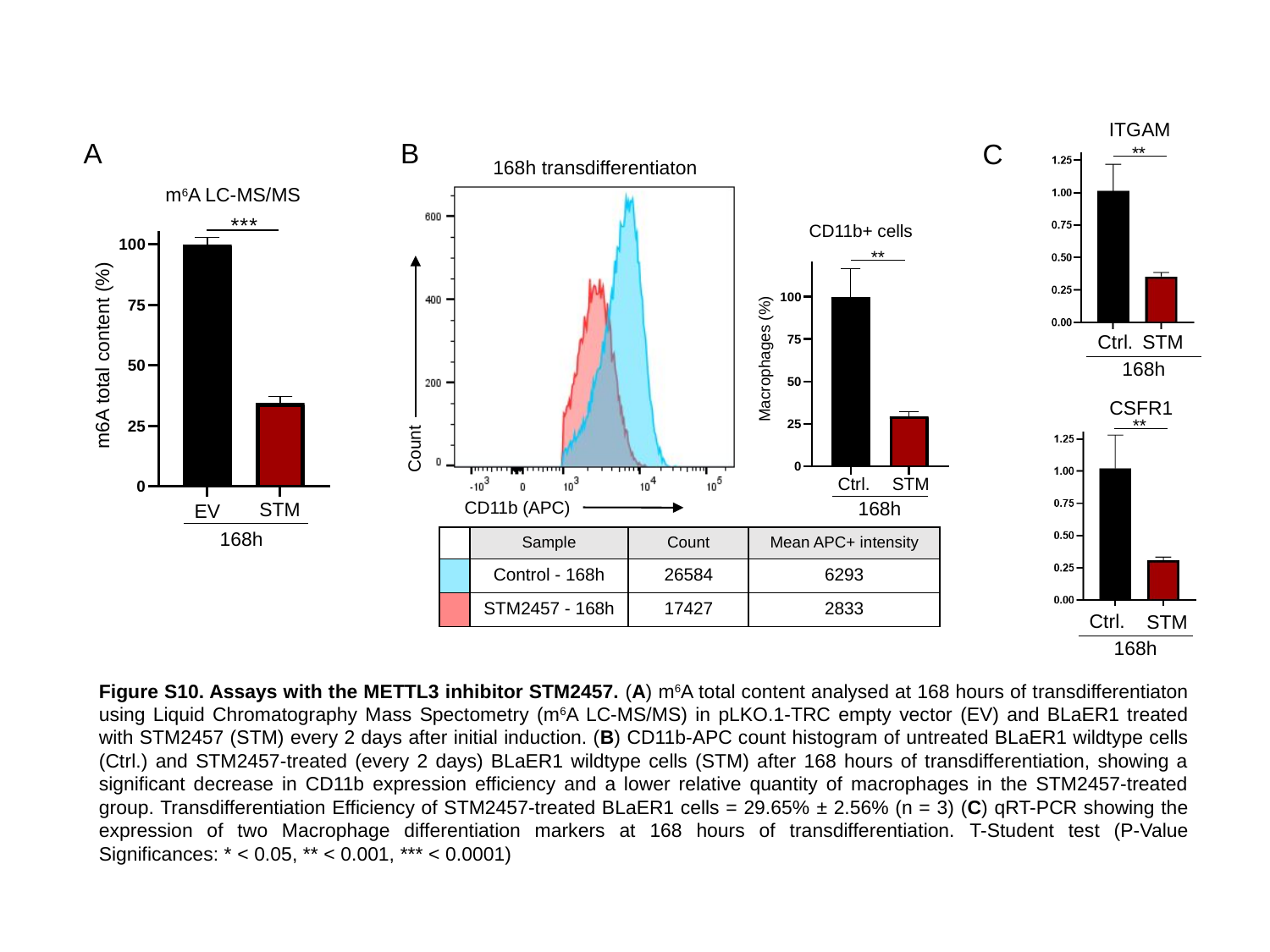

ITGAM
A
B
C
**
168h transdifferentiaton
CD11b+ cells
**
Macrophages (%)
Count
STM
Ctrl.
168h
CD11b (APC)
m6A LC-MS/MS
m6A total content (%)
STM
EV
168h
Ctrl.
STM
168h
CSFR1
**
| | Sample | Count | Mean APC+ intensity |
| --- | --- | --- | --- |
| | Control - 168h | 26584 | 6293 |
| | STM2457 - 168h | 17427 | 2833 |
Ctrl.
STM
168h
Figure S10. Assays with the METTL3 inhibitor STM2457. (A) m6A total content analysed at 168 hours of transdifferentiaton using Liquid Chromatography Mass Spectometry (m6A LC-MS/MS) in pLKO.1-TRC empty vector (EV) and BLaER1 treated with STM2457 (STM) every 2 days after initial induction. (B) CD11b-APC count histogram of untreated BLaER1 wildtype cells (Ctrl.) and STM2457-treated (every 2 days) BLaER1 wildtype cells (STM) after 168 hours of transdifferentiation, showing a significant decrease in CD11b expression efficiency and a lower relative quantity of macrophages in the STM2457-treated group. Transdifferentiation Efficiency of STM2457-treated BLaER1 cells = 29.65% ± 2.56% (n = 3) (C) qRT-PCR showing the expression of two Macrophage differentiation markers at 168 hours of transdifferentiation. T-Student test (P-Value Significances: * < 0.05, ** < 0.001, *** < 0.0001)
