# Supplementary material for: Remodeling of the m6A RNA landscape in the conversion of acute lymphoblastic leukemia cells to macrophages
Source: Leukemia. 2022 Jun 9;36(8):2121–4. doi: 10.1038/s41375-022-01621-1 (PMC9343246; doi:10.1038/s41375-022-01621-1)
Supplement: Supplementary file 11 — Supplementary Figure S11 [file 41375_2022_1621_MOESM11_ESM.pptx]

## Slide 1
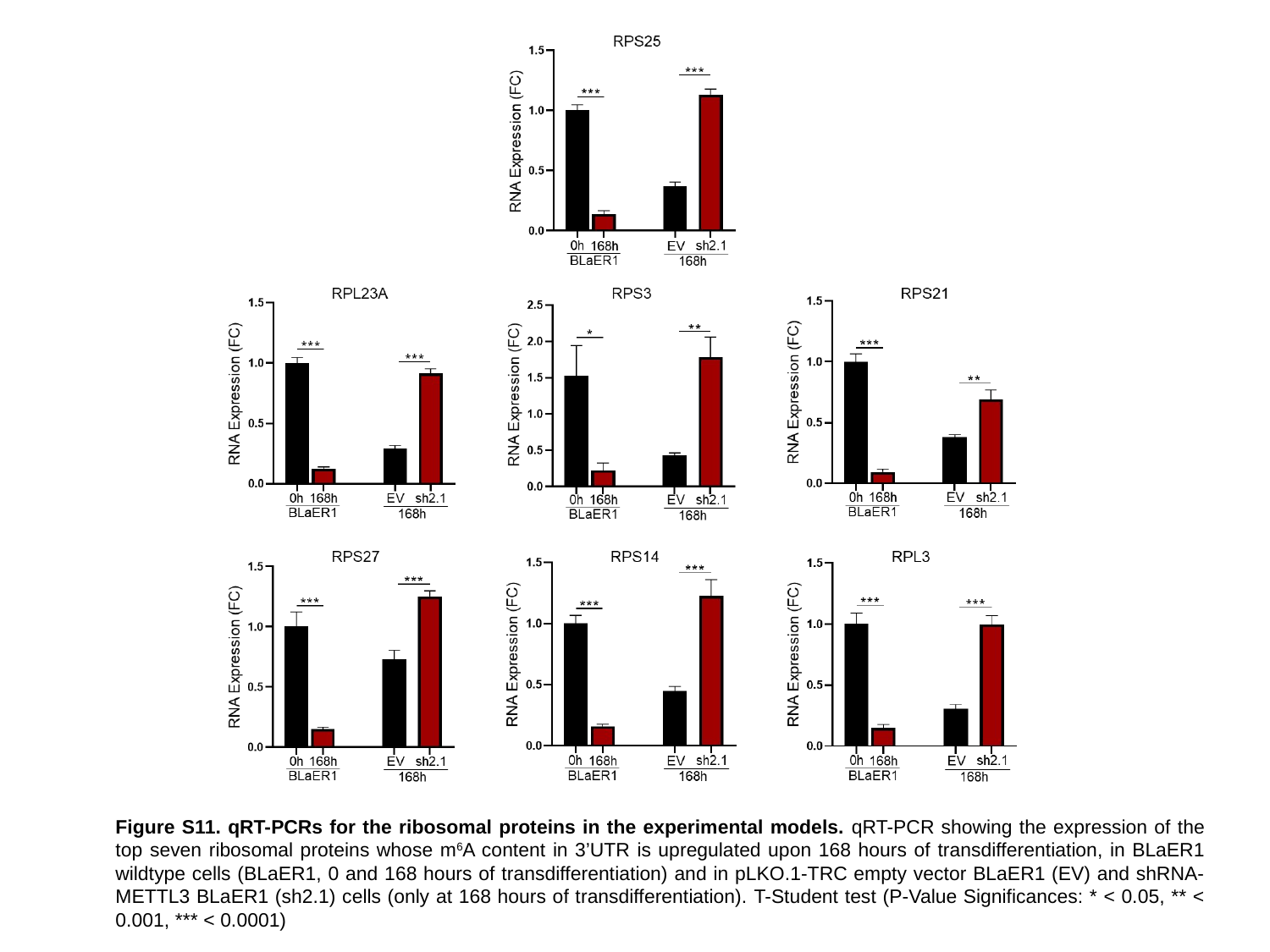

Figure S11. qRT-PCRs for the ribosomal proteins in the experimental models. qRT-PCR showing the expression of the top seven ribosomal proteins whose m6A content in 3’UTR is upregulated upon 168 hours of transdifferentiation, in BLaER1 wildtype cells (BLaER1, 0 and 168 hours of transdifferentiation) and in pLKO.1-TRC empty vector BLaER1 (EV) and shRNA-METTL3 BLaER1 (sh2.1) cells (only at 168 hours of transdifferentiation). T-Student test (P-Value Significances: * < 0.05, ** < 0.001, *** < 0.0001)
