# Supplementary material for: Remodeling of the m6A RNA landscape in the conversion of acute lymphoblastic leukemia cells to macrophages
Source: Leukemia. 2022 Jun 9;36(8):2121–4. doi: 10.1038/s41375-022-01621-1 (PMC9343246; doi:10.1038/s41375-022-01621-1)
Supplement: Supplementary file 12 — Supplementary Figure S12 [file 41375_2022_1621_MOESM12_ESM.pptx]

## Slide 1
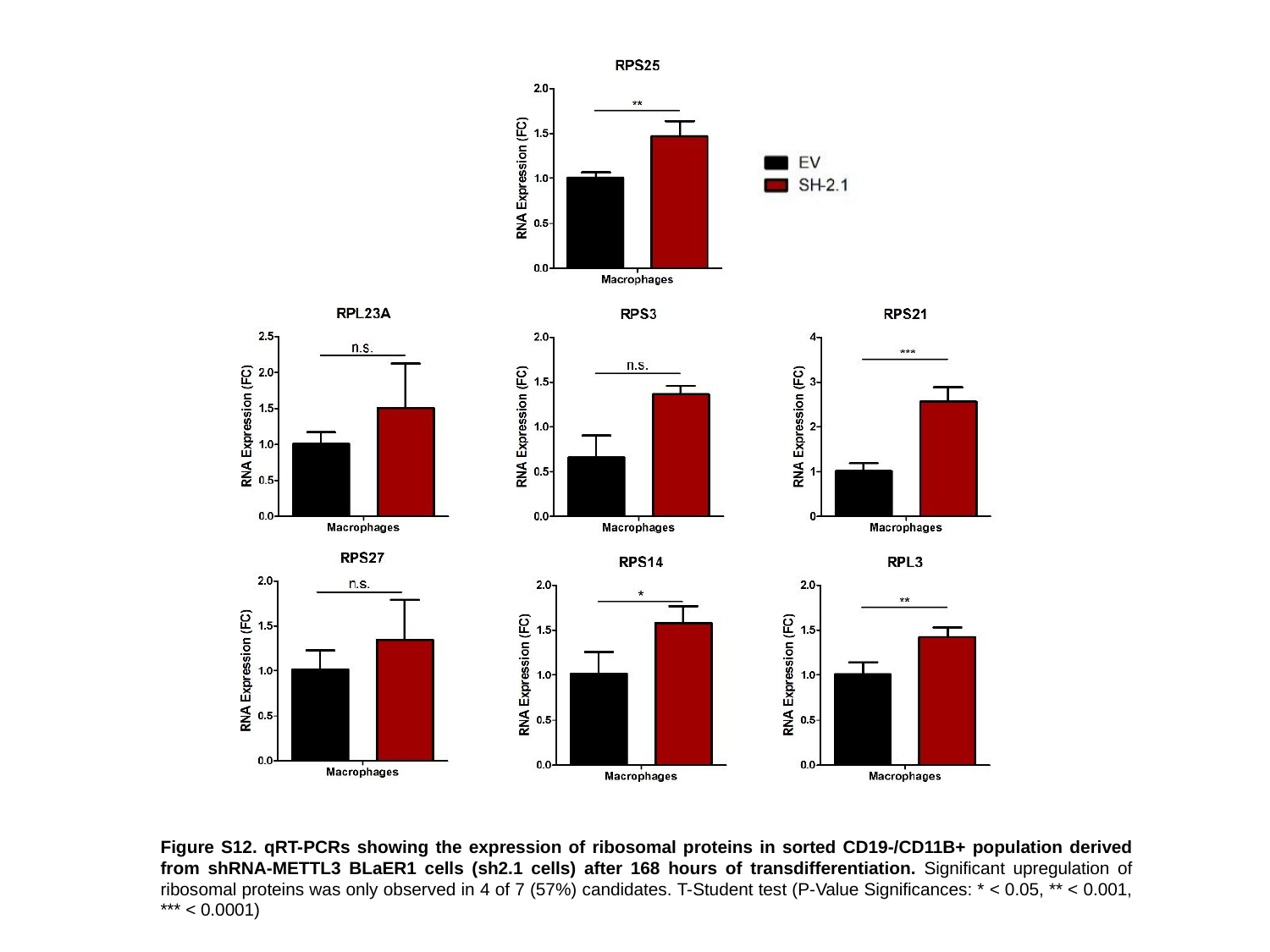

Figure S12. qRT-PCRs showing the expression of ribosomal proteins in sorted CD19-/CD11B+ population derived from shRNA-METTL3 BLaER1 cells (sh2.1 cells) after 168 hours of transdifferentiation. Significant upregulation of ribosomal proteins was only observed in 4 of 7 (57%) candidates. T-Student test (P-Value Significances: * < 0.05, ** < 0.001, *** < 0.0001)
