# Supplementary material for: Remodeling of the m6A RNA landscape in the conversion of acute lymphoblastic leukemia cells to macrophages
Source: Leukemia. 2022 Jun 9;36(8):2121–4. doi: 10.1038/s41375-022-01621-1 (PMC9343246; doi:10.1038/s41375-022-01621-1)
Supplement: Supplementary file 13 — Supplementary Figure S13 [file 41375_2022_1621_MOESM13_ESM.pptx]

## Slide 1
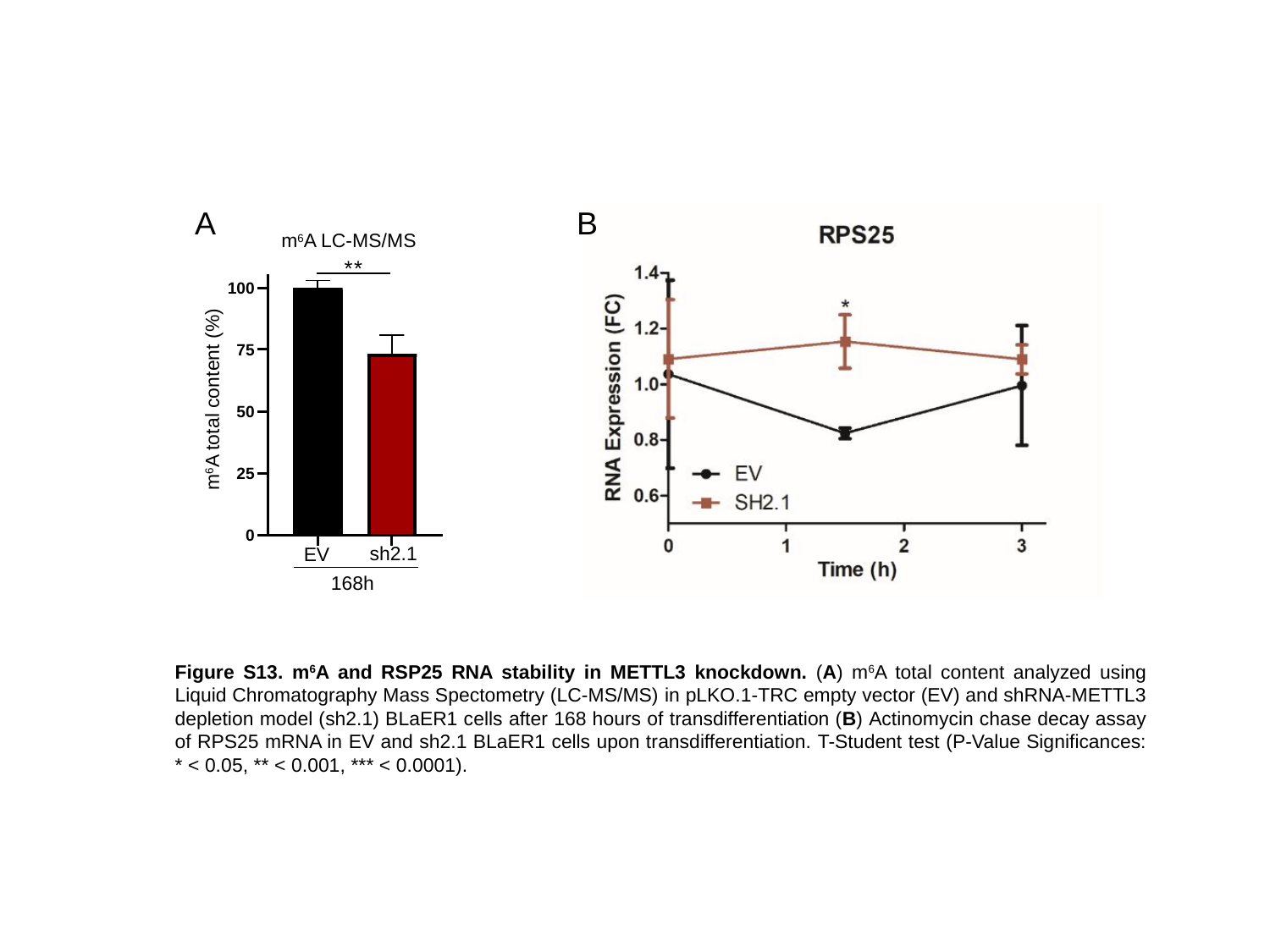

A
B
m6A LC-MS/MS
m6A total content (%)
sh2.1
EV
168h
Figure S13. m6A and RSP25 RNA stability in METTL3 knockdown. (A) m6A total content analyzed using Liquid Chromatography Mass Spectometry (LC-MS/MS) in pLKO.1-TRC empty vector (EV) and shRNA-METTL3 depletion model (sh2.1) BLaER1 cells after 168 hours of transdifferentiation (B) Actinomycin chase decay assay of RPS25 mRNA in EV and sh2.1 BLaER1 cells upon transdifferentiation. T-Student test (P-Value Significances: * < 0.05, ** < 0.001, *** < 0.0001).
